# Tree peony variegated flowers show a small insertion in the *F3’H* gene of the acyanic flower parts

**DOI:** 10.1186/s12870-020-02428-x

**Published:** 2020-05-12

**Authors:** Yanzhao Zhang, Yanwei Cheng, Shuzhen Xu, Huiping Ma, Jianming Han, Yan Zhang

**Affiliations:** 1 Life Science Department, Luoyang Normal University, Luoyang, 471022 People’s Republic of China; 2 Luoyang Research Institute of Peony, Luoyang, 471022 People’s Republic of China

**Keywords:** Tree peony, Variegated flowers, Transcriptome, Anthocyanins, *flavonoid 3′-hydroxylase* (*F3’H*) gene

## Abstract

**Background:**

The tree peony (*Paeonia suffruticosa* Andr.) cultivar ‘Er Qiao’ is appreciated for its unstable variegated flower coloration, with cyanic and acyanic flowers appearing on different branches of the same plant and occasionally in a single flower or petal. However, the variegation mechanism is still unclear.

**Results:**

In this study, we found significantly higher contents and more diverse sets of anthocyanins in the cyanic petals than in the acyanic petals. Comparative transcriptome analysis between the two flower types revealed 477 differentially expressed genes (DEGs). Quantitative real-time PCR results verified that the transcript levels of the *flavonol synthase* (*FLS*) gene were significantly increased in the acyanic petals. Furthermore, we found that a GCGGCG insertion at 246 bp in the *flavonoid 3′-hydroxylase* (*F3’H*) gene-coding region constitutes a duplication of the 241–245 bp section and was consistently found only in acyanic flowers. Sequence alignment of the *F3’H* gene from different plant species indicated that only the acyanic petals of ‘Er Qiao’ contained the GCGGCG insertion. The transformation of *Arabidopsis tt7–1* lines demonstrated that the ectopic expression of *F3’H-cyanic*, but not *F3’H-acyanic*, could complement the colors in the hypocotyl and seed coat.

**Conclusion:**

In summary, we found that an indel in *F3’H* and the upregulation of *FLS* drastically reduced the anthocyanin content in acyanic petals. Our results provide molecular candidates for a better understanding of the variegation mechanisms in tree peony.

## Background

**Fig. 1:**
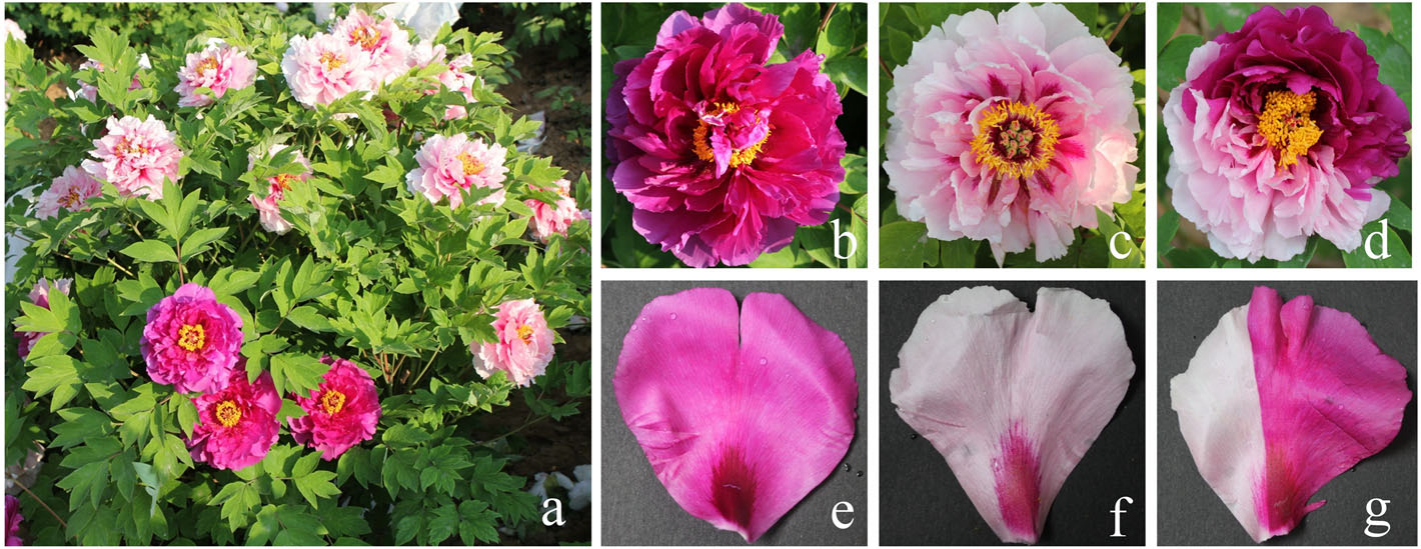
Flower coloration of *P. suffruticosa* cv. ‘Er Qiao.’ **a**. ‘Er Qiao’ plant bearing cyanic and acyanic flowers at the bloom stage; **b**–**d**. cyanic, acyanic, and variegated flowers; **e**–**g**. petals of the cyanic, acyanic, and variegated flowers

Tree peony (*Paeonia* L., Paeoniaceae) in section Moutan DC. is a perennial woody plant that exhibits a range of flower colors. Due to its high ornamental value, tree peony is considered an important horticultural flower in China. Tree peony has been cultivated in China for 1600 years, and approximately 2000 cultivars have been developed [1, 2]. Flower color is one of the most important traits in tree peony, as it dictates the ornamental value of the plant in the marketplace and is generally also used for varietal classification [3]. ‘Er Qiao’ is an ancient variety of peony that was clearly described in the 900-year-old book “Luoyang peony” written by Shihou Zhou during the Song dynasty. ‘Er Qiao’ typically produces different branches with cyanic and acyanic flowers in a single plant (Fig. 1), producing variegated flowers with cyanic and acyanic somatic sectors in a few cases. The coloration of the variegated flowers varies as follows: half cyanic and half acyanic, or with small sections of cyanic or acyanic. It is a valued cultivar due to its unstable variegated coloration, and it gained the attention of scientists due to its unique variegation characteristics [4–6]. However, the genetic mechanisms responsible for the variegated flower color of ‘Er Qiao’ have not yet been elucidated.

Flower color variegation is usually associated with differences in anthocyanin content. The anthocyanin biosynthesis pathway has been well characterized in many plants [7–9]. The substrates malonyl-CoA and 4-coumaroyl-CoA are sequentially converted into anthocyanins by the enzymes chalcone synthase (CHS), chalcone isomerase (CHI), flavanone 3-hydroxylase (F3H), dihydroflavonol 4-reductase (DFR), and anthocyanidin synthase (ANS). The action of the flavonoids 3′,5′-hydroxylase (F3’5’H) and 3′-hydroxylase (F3’H) on intermediate substrates within this pathway yields delphinidin (3′4’ 5′-hydroxylation) and cyanidin (3′4’-hydroxylation), respectively, rather than pelargonidin (4′-hydroxylation) anthocyanidins. Methylation of the 3′-hydroxyl of cyanidin produces the anthocyanidin peonidin. The anthocyanidins are converted into anthocyanins by glycosylation via uridine diphosphate -glucose: flavonoid-3-O-glycosyltransferases (UFGTs). Anthocyanins are then transported into the vacuole, where they are stored and fulfill important functions such as flower pigmentation. The MYB-bHLH-WD40 complex plays a central role in regulating the anthocyanin biosynthetic pathway, and many signals regulate anthocyanin accumulation by influencing the transcription of regulatory genes from the MYB, bHLH, and WD40 families [10, 11]. Variegated phenotypes have been observed in many plants, including morning glory [12], *Torenia* [13], and peach [14, 15], and variegation is most frequently displayed as blotches, streaks, stripes, or as different petal or leaf margin color s[16]. The gene mutations associated with flower variegation have been investigated in many plants, and research suggests that a transposable element insertion in the genes associated with anthocyanin synthesis, transport, or regulation is largely responsible [16–18]. DNA methylation and RNA interference also influence color variegation; for example, the short interfering RNA of CHS in the unpigmented sector resulted in a star-type red and white bicolor pattern in petunia [19], while methylation in the MYB10 promoter led to variable color patterning in apple skin [20]. Recently, a small indel in an anthocyanin transport gene was found to lead to variegated coloration in peach flowers [21].

The large genome of peony and the complexity of anthocyanin pathways make identifying the key genes responsible for variegation using traditional methods challenging. Transcriptome sequencing constitutes an efficient method for identifying differentially expressed genes (DEGs). Several studies have used next-generation sequencing technology to investigate the genes associated with color accumulation in tree peony [22–24]. In this study, the transcriptomes of the cyanic and acyanic petals of ‘Er Qiao’ were sequenced using the Illumina HiSeq2000 platform. Comparative transcriptome analysis and anthocyanin-related gene analysis were performed with the aim of discovering the candidate genes involved in flower variegation in tree peony.

## Results

### Anthocyanin composition in ‘Er Qiao’ flowers

**Fig. 2:**
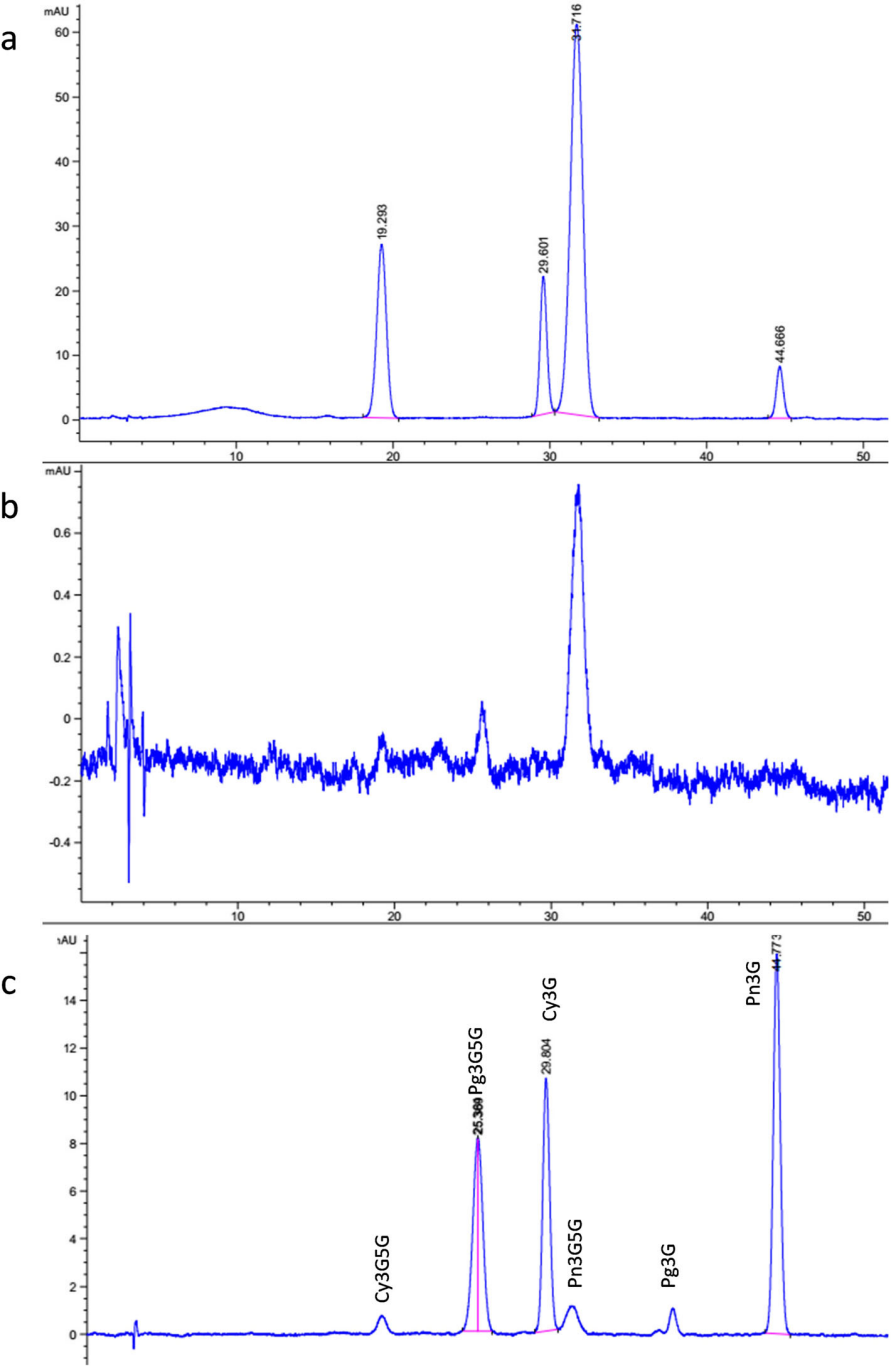
Qualitative and quantitative analysis of flower pigments using HPLC. A spot at the base of the petals was removed. **a**. HPLC chromatograms of the cyanic petals; **b**. HPLC chromatograms of the acyanic petals; **c**. HPLC chromatograms of the anthocyanin standards, including Cy3G, Cy3G5G, Pn3G, Pn3G5G, Pg3G, and Pg3G5G

High-performance liquid chromatography (HPLC) analysis was used to investigate the differences in anthocyanin content of different flower parts (Fig. 2). The anthocyanins were quantified, and the results are shown in Table 1. In the cyanic petals, the main anthocyanins were cyanidin-3,5-di-*O*-glucoside (Cy3G5G), cyanidin-3-*O*-glucoside (Cy3G), peonidin-3,5-di-*O*-glucoside (Pn3G5G), and pelargonidin-3-*O*-glucoside (Pn3G). Pn3G5G was the most dominant (2244 μg/g), followed by Cy3G5G (392 μg/g), Cy3G (93 μg/g), and Pn3G (37 μg/g). In the acyanic flowers, only Pn3G5G was detected and accounted for approximately 24.3 μg/g (92.3-fold lower than in the cyanic flowers). In addition, a very low level of pelargonidin-3,5-di-*O*-glucoside (Pg3G5G) was detected in both the cyanic and acyanic petals, and the peak area of Pg3G5G was not significantly different between the cyanic and acyanic petals. The total anthocyanin content in the cyanic petals was far higher than in the acyanic petals. In the spot region, Pn3G5G and Cy3G5G were the most dominant components, accounting for 2427 μg/g and 1201 μg/g, respectively.

**Table 1:** Composition and quantification of the anthocyanins of ‘Er Qiao’ flowers

Sample	Anthocyanin content (μg/g)					
	Cy3G	Cy3G5G	Pg3G	Pg3G5G	Pn3G	Pn3G5G
Acyanic	–	–	–	+	–	24.3
Cyanic	93	392	–	+	37	2244
Spot	84	1201	–	+	30	2427

-- indicates not detected, + indicates very low-level content

### Transcriptome sequencing and assembly

Petals from three independent plants were sampled and used for cDNA library construction. Following data filtering, 9.34, 9.31, and 9.27 Gb high-quality data were generated from the three acyanic libraries, and 9.35, 9.98, and 9.32 Gb high-quality data were generated from the three cyanic libraries. The number of clean reads with high Q20 scores was greater than 96.8%, and the GC percentage was about 45% in each of the six datasets. Each of the six sequencing results was de novo assembled, and the generated contigs, unigene numbers, and quality are indicated in Table S1. After removing redundant genes using Tigal, a total of 75,669 unigenes with 80,664,445 total residues were generated, and the average length of the unigenes was 1066 nt and the N50 was 1679 nt. A total of 28,647 unigene sequences (37.9%) had a length between 200 and 500 nt; 16,973 unigenes (22.4%) were between 500 and 1000 nt in length; 11,460 unigenes (15.1%) were between 1000 and 1500 nt in length; 7800 unigenes (10.3%) were between 1000 and 1500 nt in length; and 10,789 unigenes (14.3%) were longer than 2000 nt. Of the assembled unigenes, 43,832 were annotated in public databases.

### DEGs in acyanic and cyanic petals

Comparative transcriptome analysis showed that there were 477 DEGs between the acyanic and cyanic petals (Figure S1). In the cyanic petals, 131 unigenes were significantly upregulated, while 346 unigenes were downregulated in comparison to the acyanic petals. The DEGs were subsequently analyzed in the KEGG pathway database, and all of the 477 DEGs were assigned to 71 KEGG pathways, 18 of which were significantly enriched with Q-values ≤ 0.05 (Table S2). Within significantly enriched pathways, the largest number of sequences were those associated with metabolic pathways (58, 30.21%), followed by sequences that were involved in the biosynthesis of secondary metabolites (44, 22.92%) and phenylpropanoid biosynthesis (15, 7.81%). In particular, three flower color-related pathways, including “flavonoid biosynthesis” (ko00941), “flavone and flavonol biosynthesis” (ko00944), and “isoflavonoid biosynthesis” (ko00943), were significantly enriched. A *flavonol synthase* (*FLS*) gene, directing anthocyanin routing towards flavonols, was identified from the three color-related pathways based on the annotation, sequence alignment, and phylogenetic analysis. The key genes involved in the steps of the anthocyanin biosynthesis pathway were not found within the DEGs.

### Transcription level analysis of the candidate genes involved in flower color

**Fig. 3:**
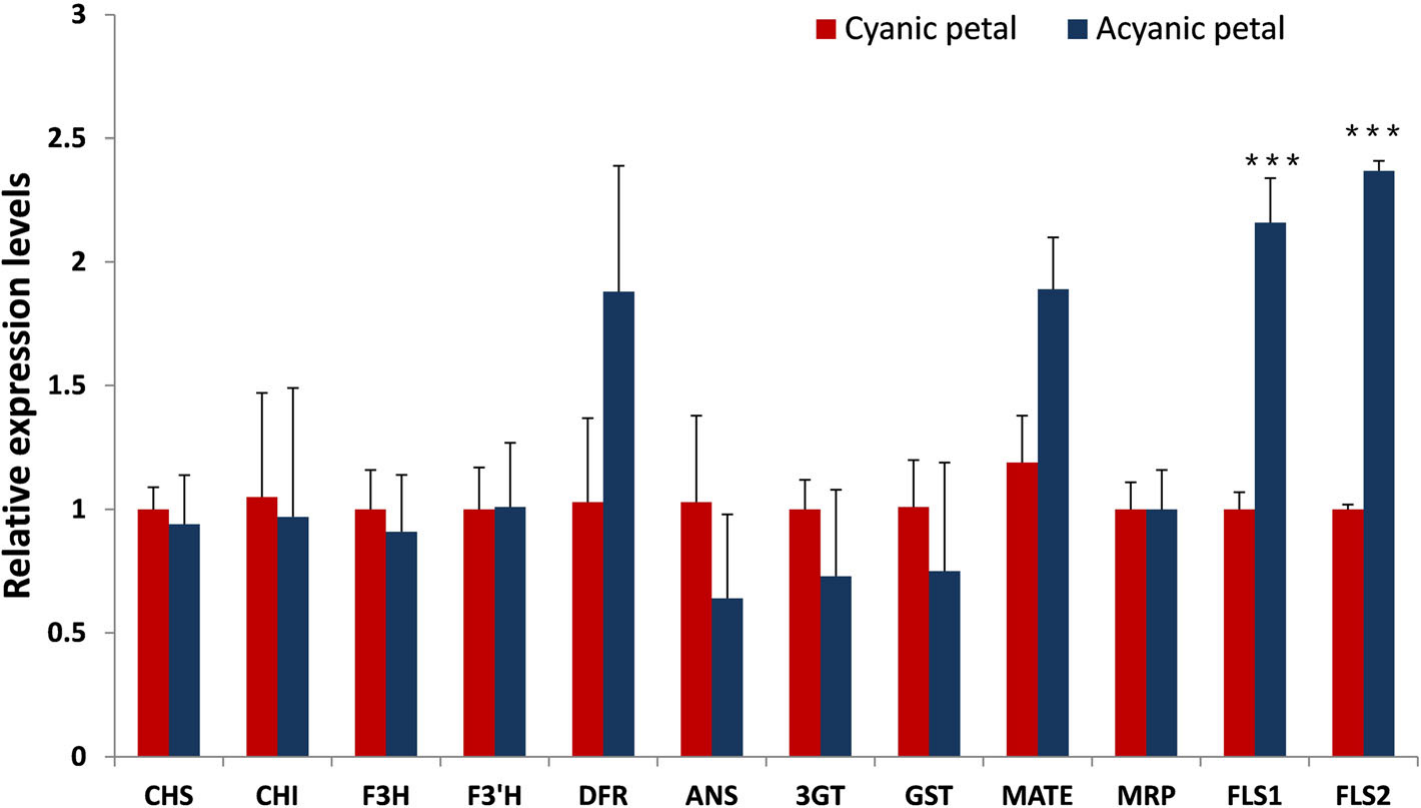
Expression level verification for candidate genes related to flower pigments using qRT-PCR. The *x*-axis indicates the gene name in the cyanic and acyanic flower samples, and the *y*-axis shows the relative gene expression levels analyzed by qRT-PCR. *Ubiquitin* was used as an internal control. The gene relative abundance represents the mean of three biological replicates. Bars indicate ± standard error. *indicates statistical significance among the cyanic and acyanic petals, as judged by *t* tests (^***^P < 0.001)

Based on the assembled unigenes, we identified two *FLS* genes and 21 genes involved in anthocyanin biosynthesis, transport, and regulation (Table 2). The fragments per kilobase of exon model per million reads mapped (FPKM) values indicated that there was only one *FLS1* gene that was significantly transcriptionally changed between the two flower types. The transcript levels of two *FLS* genes and 10 anthocyanin biosynthesis genes were evaluated using quantitative real-time (qRT)-PCR to verify the transcriptome data. Results showed that *FLS1* and *FLS2* were more significantly highly expressed in the acyanic flower petals than in the cyanic petals (*P* < 0.001) (Fig. 3). The significant upregulation of *FLS1* was confirmed by both analytical techniques, whereas the transcription level of *FLS2* was not found to be obviously changed in the transcriptome sequencing analysis. In general, the qRT-PCR results were in accordance with the transcriptome sequencing data.

**Table 2:** Identification of anthocyanin-related genes from the assembled unigenes

Gene name	Gene ID	Fpkm-acyanic	Fpkm-cyanic	Log2Ratio
Anthocyanin structural genes				
CHS	CL1003.Contig7_All	6230	6246	0.00381
CHI	Unigene23465_All	0.69	0.72	0.06
	Unigene23474_All	2.55	2.69	0.07
F3H	Unigene19708_All	13.7	16.6	0.27
	Unigene37624_All	2.4	2.4	0.0048
F3’H	Unigene27135_All	113.8	116.6	0.035
DFR	Unigene10743_All	512.7	276.3	− 0.89
ANS	CL6197.Contig1_All	6.5	12.2	0.91
	CL6197.Contig2_All	6.2	10.7	0.78
3GT	Unigene13582_All	190.2	247.9	0.38
	Unigene17020_All	0.6	0.7	0.27
5GT	Unigene15204_All	11.03	8.0	−0.46
	Unigene19312_All	20.3	8.3	−1.27
GST (tt19)	Unigene1567_All	114.07	166.5	0.55
MRP	CL7156.Contig1_All	128.4	139	0.11
MATE (TT12)	Unigene15334_All	2.8	2.0	−0.48
MYB	CL6473.Contig2_All	2.1	2.3	0.16
MYB	CL6473.Contig1_All	6.3	5.8	−0.1
bHLH	CL9074.Contig1_All	10.6	11.5	0.11
WD40	CL7979.Contig1_All	13.2	15.9	0.27
WD40	Unigene1652_All	39.2	42.2	0.1
FLS1	CL7001.Contig1_All	178.5	78	1.2
FLS2	CL7001.Contig2_All	208	162	0.35

### Sequence comparisons of anthocyanin-related transcripts between acyanic and cyanic petals

Among the six samples, anthocyanin-related transcripts were extracted from the independent assemblies and analyzed. Single nucleotide polymorphism and simple sequence repeat polymorphisms were analyzed in the 21 anthocyanin-related genes, but none were consistently identified between the two sample types. Using indel analysis, a small insertion of GCGGCG was found at the 247 bp position in the *flavonoid 3′-hydroxylase* (*F3’H*) gene, which is a duplication of its 5′ flanking fragment (241 bp to 246 bp). The small insertion was present in all of the three acyanic samples, but was absent from the three cyanic samples.

A pair of primers flanking the GCGGCG insertion was designed to amplify the *F3’H* cDNA fragment. Five independent plants with both cyanic and acyanic branches were selected. Their cDNAs were used as templates, and the PCR products were sequenced. The results showed that all the acyanic petals contained the GCGGCG insertion, which was absent in the cyanic petals (Figure S2). Furthermore, in three flowers with both cyanic and acyanic petals, the insertion was only present in the acyanic petals. Our results suggest that the GCGGCG insertion in the *F3’H* gene is correlated with acyanic petals in ‘Er Qiao.’

The *F3’H* fragment was amplified in the genomic DNA using PCR, and 20 clones from each sample were selected and sequenced. Using three biological replicates, we found that both the normal sequence and the sequence with 6-bp insertion fragments were present in the cyanic and acyanic petals.

### *F3’H* gene analysis

**Fig. 4:**
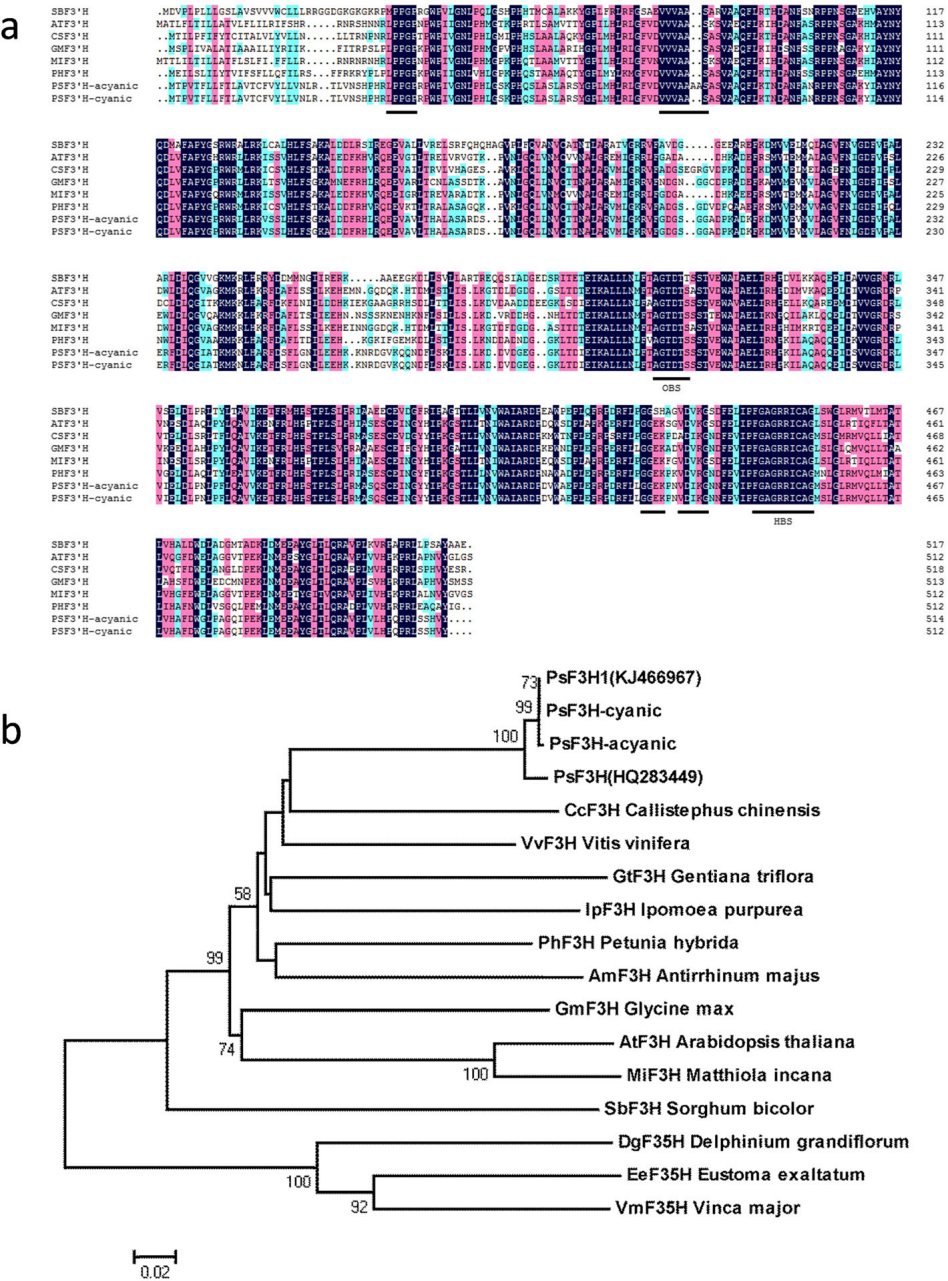
Sequence characterization and phylogenetic analysis. **a**. Multiple sequence alignment of the deduced amino acid sequences of F3’H from ‘Er Qiao’ cDNA and six other plant species was conducted using DNAMAN software. Sequences used are from *A. thaliana* (AF271651), *Callistephus chinensis* (AF313488), *Glycine max* (AF499731), *Matthiola incana* (AF313491), *Petunia hybrida* (AF155332), and *Sorghum bicolor* (AAV74195). **b**. Phylogenetic tree showing the evolutionary relatedness of ‘Er Qiao’ F3’H with that of other plants. The accession numbers of the included F3’H proteins are as follows: *P. hybrida* (X71130), *Antirrhinum majus* (AB028151), *Gentiana triflora* (BAD91808), *Ipomoea purpurea* (AAR00229), *Matthiola incana* (AAG49301), *Vitis vinifera* (BAE47006), *Delphinium grandiflorum* (AAX51796), *Eustoma exaltatum* (BAD34460), *Vinca major* (BAC97831), *Paeonia suffruticosa* (HQ283449), and *Paeonia suffruticosa* (KJ466967). The tree was constructed using the neighbor-joining method in MEGA5 software. Numbers along the branches indicate bootstrap support determined from 1000 repetitions, and the bar indicates an evolutionary distance of 0.02%

To verify the gene sequence results obtained by transcriptome sequencing, the *F3’H* cDNA was amplified using PCR and cloned into a pMD19-T vector. Ten clones were sequenced, and the results showed that the gene sequences were identical to that of the de novo assembly. Amino acid sequence alignment of F3’H with that from other plant species is shown in Fig. 4a. The F3’H-cyanic was identical to those of the tree peony cultivar “Luoyanghong” [25] and showed 60–73% similarity with other F3’Hs from dicots and monocots. It had the highest similarity with *Callistephus chinensis* at 73%. Domains that are conserved among many cytochrome P450s, including heme- and oxygen-binding sites, were also found in the F3’H of tree peony. Three F3’H-specific conserved motifs, namely VDVKG, VVVAAS, and GGEK, were identical to those previously reported [26, 27]. While for F3’H-acyanic, two alanine insertions caused by the GCGGCG insertion altered the F3’H specific motif from VVVAAS to VVVAAAAS, which was only present in the acyanic flowers of ‘Er Qiao.’ Phylogenetic analysis was performed based on the deduced amino acid sequences of F3’H and F3’5’H, which have diverse functions in plants (Fig. 4b). The phylogenetic tree indicated that the two F3’H proteins of tree peony clustered in a clade with the F3’H proteins from both dicots and monocots.

We also cloned and sequenced the *dihydroflavonol 4-reductase* (*DFR*) gene from cDNAs of cyanic and acyanic petals. The results showed that the *DFR* sequence was identical in the two samples.

### Functional analysis of *PsF3’H* genes in the *Arabidopsis* mutant *tt7–1*

**Fig. 5:**
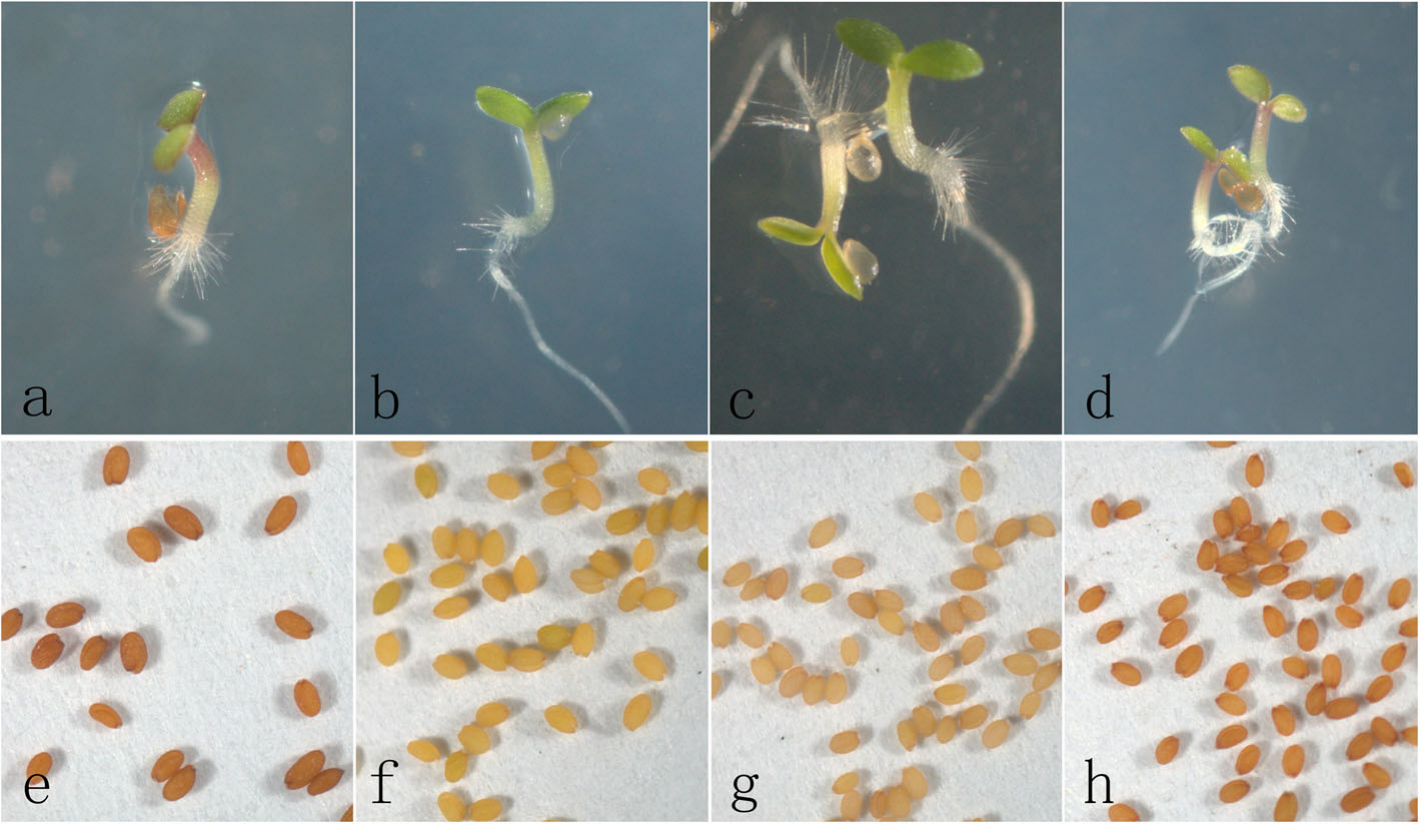
Complementation of the pigmentation of *Arabidopsis tt7* mutant with *PsF3’H* genes. **a**–**d**, phenotypes of wild-type, *tt7–1*, *F3’H-acyanic,* and *F3’H-cyanic* transgenic line seedlings; **e**–**h**, phenotypes of wild-type, *tt7–1*, *F3’H-acyanic*, and *F3’H-cyanic* transgenic line seeds

The *Arabidopsis transparent testa7–1* (*tt7–1*) mutant does not have a functional *F3’H* gene. The seed coat is pale brown due to the disruption of the proanthocyanidin pathway, and the epicotyl is pale green due to the disruption of the anthocyanin pathway. To compare functions of the two *PsF3’H* allelic genes, *PsF3’H-cyanic* and *PsF3’H-acyanic* were separately ligated to the cauliflower mosaic virus 35S promoter and transferred into the *Arabidopsis tt7–1* mutant. More than five transgenic plants were produced for each of the *PsF3’H* allelic genes. The germinating seedlings of wild-type, *tt7–1* mutant, *PsF3’H-acyanic,* and *PsF3’H-cyanic* transgenic lines are shown in Fig. 5a–d, and the seeds of the wild-type, *tt7–1* mutant, *PsF3’H-acyanic,* and *PsF3’H-cyanic* transgenic lines are shown in Fig. 5e–h. Wild-type and *PsF3’H-cyanic* transgenic lines had red hypocotyls, but *Arabidopsis tt7–1* mutant and *PsF3’H-cyanic* transgenic lines had pale green hypocotyls.

Moreover, seeds collected from transgenic lines expressing *PsF3’H-cyanic* showed the pigmentation characteristic of the wild-type *Arabidopsis*, while the seeds of the *Arabidopsis tt7–1*mutant and transgenic lines expressing *PsF3’H-acyanic* were pale brown in color.

## Discussion

The strikingly low level of anthocyanins in the acyanic petals of ‘Er Qiao’ as compared to the cyanic petals suggests limited activity of the anthocyanin pathway in the acyanic petals. The transcription levels of genes in the anthocyanin pathway directly determine anthocyanin content and composition. It has been reported that the downregulation of some of these genes leads to a decrease in anthocyanin accumulation in peach [15], *Linaria* [28], Japanese morning glory [12], and Japanese apricot [18]. However, in the present study, differential transcript levels of key genes in each step of the anthocyanin pathway were not found between the two flower samples of ‘Er Qiao.’ Therefore, the varied pathway activity may not be related to the differential transcription levels of genes in the anthocyanin pathway. *FLS* encodes a flavonol synthase and could affect plant pigment. Its substrate dihydroflavonols compete with *DFR*, *F3’H* and *F3’5’H* in the anthocyanin pathway, directing the route towards flavonols. In *Arabidopsis*, anthocyanin content is reduced in *FLS* over-expression lines but increased in the *fls* mutant [29, 30]. In this study, we found that an *FLS1* gene was differentially expressed and was upregulated 2.1-fold in the acyanic petals. The *FLS2* gene was also verified as upregulated in the acyanic petals by qRT-PCR. We deduced that the higher transcription level of *FLS* acyanic petals may redirect more dihydroflavonols towards the biosynthesis of flavonols, and the lack of substrate will reduce the biosynthesis of anthocyanins, which may be one important reason for the reduction in petal color.

Small indels in coding regions can affect the function of a gene but not alter its transcription level. As observed in the peach cultivar “Hongbaihuatao,” subtle differences in a gene sequence, such as a small indel in the *GST* gene [21], can destroy the enzyme activity and result in color variegation. By analyzing the anthocyanin-related gene sequences from cyanic and acyanic flowers, we discovered that only a GCGGCG insertion in the *F3’H* gene could clearly distinguish the two genotypes, suggesting that the insertion may influence the flower color in ‘Er Qiao.’

The flavonoid pathway diverges into side branches by *F3’H* and *F3’5’H* and finally generates cyanidin−/peonidin -, delphinidin- and pelargonidin-derived anthocyanins*. F3’H* is a critical enzyme that determines cyanic/acyanic petal variation. It hydroxylates the β-ring of dihydrokaempferol (DHK) at the 3′ position to generate dihydroquercetin (DHQ), ultimately leading to the production of cyanidin-based pigments [31]. A mutation in the *F3’H* gene usually leads to a change in plant color; for example, a single-base deletion in the soybean *F3’H* gene resulted in a change from brown to a gray pubescence color [32]. In the morning glory species *Ipomoea nil*, *I. tricolor,* and *I. purpurea*, reddish flowers were also caused by spontaneous mutations in the *F3’H* gene [33]. Several studies have indicated that some landmark sequences, including GGEK, GGSH, VVVAAS, and VDVKG, are present in the F3’H protein [26, 27]. In this study, the GCGGCG insertion, which altered the conserved motif from VVVAAS to VVVAAAAS, was only present in the acyanic branches of ‘Er Qiao.’ The transformation of *Arabidopsis tt7–1* lines has demonstrated that the ectopic expression of *F3’H*-cyanic, but not *F3’H*-acyanic, could complement its colors in the hypocotyl and seed coat. Our results demonstrate that the small insertion in *F3’H* impairs gene function, leading to the drastic reduction or absence of cyanidin−/peonidin-type anthocyanins in the acyanic petals of ‘Er Qiao.’ Usually, the seed coat of tree peony seeds is dark brown. ‘Er Qiao’ is sterile and is generally propagated by grafting. As we did not have mature seeds, we were unable to show the relationship between the small insertion and seed coat color in this study.

Generally, a non-functional *F3’H* would turn the formation of cyanidin−/peonidin-type anthocyanins into pelargonidin-type anthocyanins and delphinidin-type anthocyanins. Previously, research showed that delphinidin-type anthocyanins and *F3’5’H* were absent in tree peony [34, 35]. In this study, delphinidin-type anthocyanins and *F3’5’H* were not also detected in ‘Er Qiao.’ Therefore, we suggest that the *F3’5’H* gene is absent in ‘Er Qiao,’ and the functional disruption of *F3’H* in acyanic petals cannot lead to delphinidin-type anthocyanin accumulation. The DFR enzyme has a substrate-specificity determining region, and as observed in petunia, substrate specific DFR did not efficiently reduce dihydrokaempferol, which is an essential step for pelargonidin production [36, 37]. The alteration of one single amino acid may be sufficient to change the substrate specificity of DFR [36]. Through the analysis of a large number of tree peony varieties with colored flowers, it was found that some varieties contained pelargonidin-type, while others did not [34, 35]. This may be related to different substrate specific *DFR* genes. In our study, the sequence of *DFR* was identical in the cyanic and acyanic flowers (Figure S3), but we did not observe changes from cyanidin−/peonidin-type to pelargonidin-type anthocyanins in the acyanic petals, which may due to low dihydrokaempferol conversion efficiency or substrate competition between DFR and FLS.

**Fig. 6:**
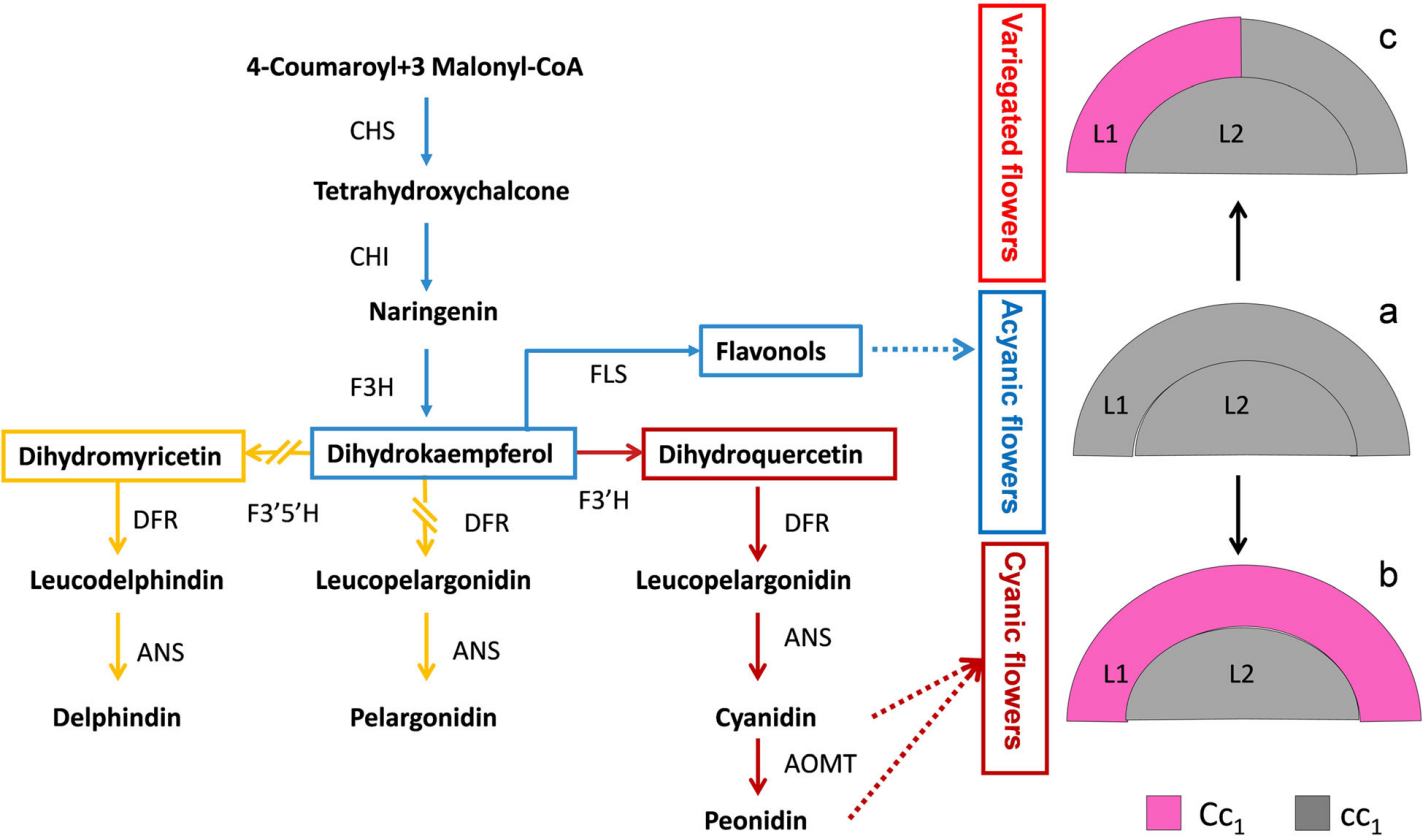
A proposed model for the flower color in ‘Er Qiao.’ Red arrows represent the main branch of the flavonoid pathway in cyanic flowers, and the blue arrows represent the main branch in acyanic flowers. The orange arrow represents a potentially very inefficient step. L1 and L2 indicate different layers of floral meristems. C represents the *F3’H-cyanic* gene, c represents the *F3’H-acyanic* gene, c_1_ represents a putative non-transcribed *f3’h* gene with no 6-bp insertion (*F3’H-acyanic2*). a. Acyanic flower carrying two nonfunctional alleles of the *F3’H* gene. b. Cyanic flower derived from a periclinal chimera. L1 layer carrying one functional *F3’H* allele. c. Variegated flower (displaying acyanic and cyanic somatic sectors) derived from a mericlinal chimera. A fraction of the L1 layer carrying one functional *F3’H* allele

Chimeras are important factors responsible for variegated flowers and are usually caused by genetic mutations in the cell division of shoot apical meristems [21]. In dicots, shoot apical meristems typically contain three cell layers: the L1 layer gives rise to the epidermis, L2 and L3 contribute to the internal tissues, and the petals are derived solely from the L1 and L2 layers [38]. According to the arrangement of genetically different cells in the shoot apical meristem, chimeras can be divided into three types, including sectorial, mericlinal, and periclinal chimeras, which dictate different plant phenotypes [16]. In the peach cultivar “Hongbaihuatao,” the white-flower branch is the principal branch, and the L1 layer contains two nonfunctional *Riant* alleles and is unable to accumulate anthocyanins [21]. A pink flower is a periclinal chimera with the L1 layer containing a functional *Riant* allele and a nonfunctional *Riant* allele. Variegated flowers displaying pink and white somatic sectors are derived from mericlinal chimeras, in which a fraction of the L1 layer contains a functional *Riant* allele and a nonfunctional *Riant* allele. In ‘Er Qiao,’ the acyanic-flower branch is the principal branch, and the cyanic-flower and variegated-flower branches are deemed to arise from bud sports. We found that the color difference between cyanic and acyanic petals was mainly in the epidermal cells, while the internal tissues did not show color (Figure S4). Combining the *F3’H* fragments from genomic DNA and cDNA, it was speculated that acyanic flowers should be heterozygous with *F3’H-acyanic* and a non-transcribed *f3’h* fragment without the 6-bp insertion (*F3’H-acyanic2*), both of which were non-functional alleles of *F3’H*. We infer that the cyanic flower is a periclinal chimera, with the L1 layer containing a functional *F3’H-cyanic* and being capable of accumulating anthocyanins. The variegated flower, displaying acyanic and cyanic somatic sectors, may be derived from a mericlinal chimera, in which a fraction of the L1 layer contains a functional *F3’H-cyanic*. Thus, a model is proposed for the variegated phenotype in the flower coloration of ‘Er Qiao’ (Fig. 6). In the model, the L2 layer of the cyanic flower contained an *F3’H-acyanic* gene, but its transcript level was not detected. In tree peony flowers, pigments mainly accumulated in the epidermal cells [39, 40], which suggests that anthocyanin synthesis genes were mainly transcribed in the epidermal cells. We suggest that the *F3’H-acyanic* gene is not transcribed or has a very low transcription level in the inner cells of ‘Er Qiao’ petals, which may explain why *F3’H-acyanic* was not detected at the transcriptional level in the cyanic petals.

In the tree peony transcriptome, the numbers of assembled unigenes in several studies differ. For example, 29,275 unigenes were assembled from the bud transcriptome [41], 66,536 unigenes were assembled from the carpel transcriptome [42], and 80,390 unigenes were assembled from the flower transcriptome [43], while in our study, a total of 75,669 unigenes were assembled from the petal transcriptome. Key candidate genes were obtained in all above studies. Therefore, we suggest that all the above assemblies were reliable, and tissue-specific difference is one reason for the differences in the number of assembled unigenes. By analyzing the 447 DEGs using the KEGG database, 18 pathways were found to be significantly enriched. This suggests that there are significant differences in intracellular metabolism between the two color petals, possibly due to the *F3 ‘H* mutation, which leads to the switch of anthocyanin synthesis pathways to other metabolic pathways. In a future study, the mining of the 477 DEGs will further clarify the relationship between different metabolic pathways and flower color, and further improve the mechanism of the formation of variegated flowers. Recently, Wang et al. [41] sequenced the tree peony bud transcriptome and assembled 29,275 unigenes. Their findings should assist in identifying the cause of *F3 ‘H* gene mutation through the mining of genes transcribed in the bud.

## Conclusions

We analyzed the gene variation in the flavonoid pathway and proposed a model for the variegated coloration of ‘Er Qiao’ flowers (Fig. 6). Diversion of the flavonoid pathway branch towards cyanidin−/peonidin-type anthocyanins through reduced *FLS* activities and a functional *F3’H* is responsible for the cyanic petals. In contrast, in the acyanic petals, the significantly increased activity of *FLS* may result in the diversion of the substrate dihydrokaempferol to form flavonols. Furthermore, a small insertion found in *F3’H* disrupts its activity, impeding cyanidin−/peonidin-type anthocyanin biosynthesis, which explains the severe reduction in pigments. We speculate that cyanic flowers are derived from periclinal chimeras, and acyanic flowers with cyanic somatic sectors are derived from mericlinal chimeras. Our results provide molecular mechanistic insights into the flower variegation trait in tree peony.

## Methods

### Tissue collection and quantification of anthocyanins

*Paeonia suffruticosa* Andr. cv. ‘Er Qiao’ is a traditional variety in China and is recorded in the book tree peony of China [44]. The plants used in this study were kindly identified and provided by senior engineer Zhengfeng Peng (Luoyang Research Institute of Peony, Luoyang, China) and were preserved in the tree peony germplasm repository at the Luoyang Research Institute of Peony (Luoyang, China). Three healthy plants displaying both cyanic and acyanic flowers on different branches were selected for research. At the soft bud stage, the spot at the base of the petals was removed from each plant, and the remaining cyanic and acyanic petals were respectively sampled and pooled.

For anthocyanin quantification, anthocyanin extraction was prepared as described by Zhang et al. [23]. The petals from each sample were ground to a powder in liquid nitrogen, and 0.5 g sample was extracted with 5 mL acidified methanol (0.05% hydrochloric acid). After centrifugation, the supernatant was filtered through a 0.22-μm membrane filter and used for detection. Agilent 1290 HPLC system (Agilent, Santa Clara, CA, USA) was used to analyzed anthocyanins, and the chromatographic analysis conditions were as described by Zhao et al. [45]. The injection volume was 20 μL. The mobile phase consisted of 10% formic acid water solution as solvent A (10:90; v/v, HCOOH:H2O) and methyl alcohol/acetonitrile/water (10:40:50; v/v/v, CH3OH:CH3CN:H2O) as solvent B. The linear gradient profile was 10% B at 0 min, 20% B at 30 min, 30% B at 50 min, 40% B at 60 min. The solvent gradient described above was applied at a flow rate of 0.8 mL/min. Six anthocyanins, including Cy3G, Cy3G5G, Pn3G, Pn3G5G, pelargonidin-3-*O*-glucoside (Pg3G), and Pg3G5G have been reported as the primary pigments in tree peony [34] and were selected for the qualitative and quantitative analysis of the anthocyanin composition of ‘Er Qiao’ flowers. Anthocyanin standards were ordered from Beijing Solarbio Science & Technology Co., Ltd. (Beijing, China). Three biological replicates were performed.

### Library construction and sequencing

Total RNA was extracted using the CTAB method and then subsequently treated with RNase-Free DNase I (Qiagen, USA) to eliminate genomic DNA. Via detection with Agilent 2100 Bioanalyzer, high-quality RNA with an integrity number > 8.5 were selected for further analysis. The poly-A-containing mRNA was purified using oligo (dT) magnetic beads, and then cleaved into small pieces using fragmentation buffer. The first cDNA strand was synthesized using random hexamer primers and reverse transcriptase, followed by second-strand cDNA synthesis using DNA polymerase I and RNase H. The short double-stranded fragments were purified with a QiaQuick PCR Purification Kit (Qiagen, CA, USA) and dissolved with the EB buffer supplied in the kit for end preparation and poly(A) addition. The sequence adaptors were linked to two ends of the short cDNA sequences. Agarose gel electrophoresis was used to select suitably sized fragments, and the products were subsequently amplified by PCR. The libraries were sequenced using an Illumina HiSeq™ 2000 platform (Illumina Inc., San Diego, CA, USA) at the Beijing Genomic Institute (Shenzhen, China). Three biological replicates were tested for transcriptome sequencing. The raw sequence data reported in this paper have been deposited in the Genome Sequence Archive [46] in BIG Data Center [47], Beijing Institute of Genomics (BIG), Chinese Academy of Sciences, under accession number CRA000698 and are publicly accessible at http://bigd.big.ac.cn/gsa.

### De novo assembly and gene annotation

We did de novo assembly and gene annotation using methods described by Zhang et al. [48]. The raw reads were filtered, and the clean reads from each of the six libraries were de novo assembled using Trinity software v. 2.0.6 [49]. Further assembly was performed using a TGI clustering tool (Tgicl) v. 2.06 [50] to generate non-redundant unigenes. To annotate the assembled unigenes, BLAST algorithm v. 2.2.23 (evalue<1e^− 5^) was used to query public databases, including the NCBI non-redundant (Nr), nucleotide (Nt), SWISS-PROT, InterPro, and Kyoto Encyclopedia of Genes and Genomes (KEGG) databases. The best hit results were used to explain gene function.

### Identification of significant DEGs

Clean reads were mapped to unigenes using Bowtie2 v. 2.2.5 [51], and gene expression levels were measured using fragments per kb per million reads (FPKM) [52]. The statistical significance of the differential expression was determined using NIOSeq software [53]. The DEGs were selected using a threshold of probability ≥0.8 and an absolute value of log2 ratio ≥ 1. Based on the KEGG and Gene Ontology (GO) annotations, enrichment analyses were performed using hypergeometric tests. A false discovery rate (FDR) < 0.05 was set as the threshold for screening the significantly enriched GO and KEGG terms.

### Expression level verification

Gene transcript levels were measured using qRT-PCR on an ABI 7500 system. Each reaction contained 5 μL of 2 × SYBR Green qPCR Master Mix, 4 μM of gene-specific primers, and 1 μL of cDNA in a final volume of 10 μL. The PCR reactions were performed under the following conditions: 95 °C for 3 min, followed by 45 cycles of 95 °C for 7 s, 57 °C for 10 s, and 72 °C for 15 s. Primers are listed in Table S3. Three biological replicates were performed for each gene. The relative expression levels of each gene were calculated using the 2^-ΔΔCt^ method [54], and the *ubiquitin* gene was used as an internal control [55, 56].

### Expression vector construction and plant transformation

A pair of primers, 5′-GACTCTTGACCATGGTAGATCTAATGACTCCCGTGACTTTTC-3′/5′-GGGAAATTCGAGCTGGTCACCCTAGTACACGTGGGATGAG-3′, was designed to amplify the coding region of the *PsF3’H*s using cDNA synthesized from flowers as templates. The plasmid pcambia1301 was digested with BglII and BstEI, and the PCR products of *F3’H-cyanic* and *F3’H-acyanic* were ligated to pcambia1301 using a Vazyme ClonExpress-II One Step Cloning Kit (Vazyme Biotech Co., Ltd). The *Arabidopsis tt7–1* mutant (CS88) was obtained from the Arabidopsis Biological Resource Center (Ohio State University, OH, USA). *Arabidopsis* transformation was performed according to the floral dip method [57]. For transgenic plant selection, T0 seeds were sterilized and germinated on Murashige and Skoog medium containing 20 mg/L Hygromycin B. The resistant lines were transplanted to the soil 10 d later and placed in a growth chamber at 25 °C and 50–80% relative humidity. The *DFR* gene was cloned using primers 5′-GTTCATCGGTTCATGGCTTGT-3/5′-TTCCA TGGGAAGTGGAAGCAA-3′. *F3’H* fragments were cloned using primers 5′-ATTCACCTTAGCGGTTACCTG-3′/5′-TGCAAACACAA GGTCCTGATA-3′.

## Supplementary information

**Additional file 1: Figure S1.** DEGs between the cyanic and acyanic petals. Transcriptome sequencing was performed with three biological replicates. The parameters “probability ≥0.8” and “log2 ratio ≥1” were used as the thresholds to determine the DEGs. An orange triangle represents the upregulated genes, a green square indicates the downregulated genes, and gray dots indicate the genes that did not change significantly between the two transcriptomes.

**Additional file 2: Figure S2.** Validation of the small insertion in the *F3’H* cDNAs. a. Fragment sequence of the F3’H cDNA in cyanic flowers; b. fragment sequence of the *F3’H* cDNA in acyanic flowers.

**Additional file 3: Figure S3.** The alignment of DFR amino acids from cyanic and acyanic branches of ‘Er Qiao.’ PsDFR (GenBank accession number KJ466968) was obtained from *Paeonia suffruticosa* cultivar ‘Luoyang Hong.’

**Additional file 4: Figure S4.** Examination of anthocyanin accumulation in the petal cell layers. Photos of epidermal cells (original magnification, 400×) and cross-sections (original magnification, 100×) were taken under a microscope.

**Additional file 5: Table S1.** Statistics of assembly quality. **Table S2.** KEGG enrichment of the DEGs between cyanic and acyanic flowers. **Table S3.** Primers used for qRT-PCR.

## Data Availability

The datasets supporting the conclusions of this article are available in the Genome Sequence Archive in BIG Data Center, Beijing Institute of Genomics (BIG), Chinese Academy of Sciences, with the accession numbers CRA000698 (https://bigd.big.ac.cn/gsa/), and in the NCBI Sequence Read Archive (SRA) under accession number PRJNA431059 (http://www.ncbi.nlm.nih.gov/sra).

## Abbreviations

ANS: Anthocyanidin synthase
BIG: Beijing Institute of Genomics
CHI: Chalcone isomerase
CHS: Chalcone synthase
Cy3G: Cyanidin-3-*O*-glucoside
Cy3G5G: Cyanidin-3,5-di-*O*-glucoside
DEGs: Differentially expressed genes
*DFR*: *Dihydroflavonol 4-reductase*
DHK: Dihydrokaempferol
DHQ: Dihydroquercetin
FDR: False discovery rate
*FLS*: *Flavonol synthase*
F3H: Flavanone 3-hydroxylase
*F3’H*: *Flavonoid 3′-hydroxylase*
*F3’5’H*: *Flavonoid 3′,5′-hydroxylase*
FPKM: Fragments per kilobase of exon model per million reads mapped
GO: Gene Ontology
HPLC: High-Performance Liquid Chromatography
KEGG: Kyoto Encyclopedia of Genes and Genomes
Nr: Non-redundant
Nt: Nucleotide
Pg3G: Pelargonidin-3-*O*-glucoside
Pg3G5G: Pelargonidin-3,5-di-*O*-glucoside
Pn3G: Peonidin-3-*O*-glucoside
Pn3G5G: Peonidin-3,5-di-*O*-glucoside
qRT-PCR: quantitative real-time PCR
Tgicl: TGI clustering tool
*tt7–1*: *transparent testa7–1*
UFGTs: Uridine diphosphate-glucose: flavonoid-3-O-glycosyltransferases
