# Supplementary figures and images for: Tree peony variegated flowers show a small insertion in the F3’H gene of the acyanic flower parts
Source: BMC Plant Biol. 2020 May 12;20:211. doi: 10.1186/s12870-020-02428-x (PMC7216414; doi:10.1186/s12870-020-02428-x)

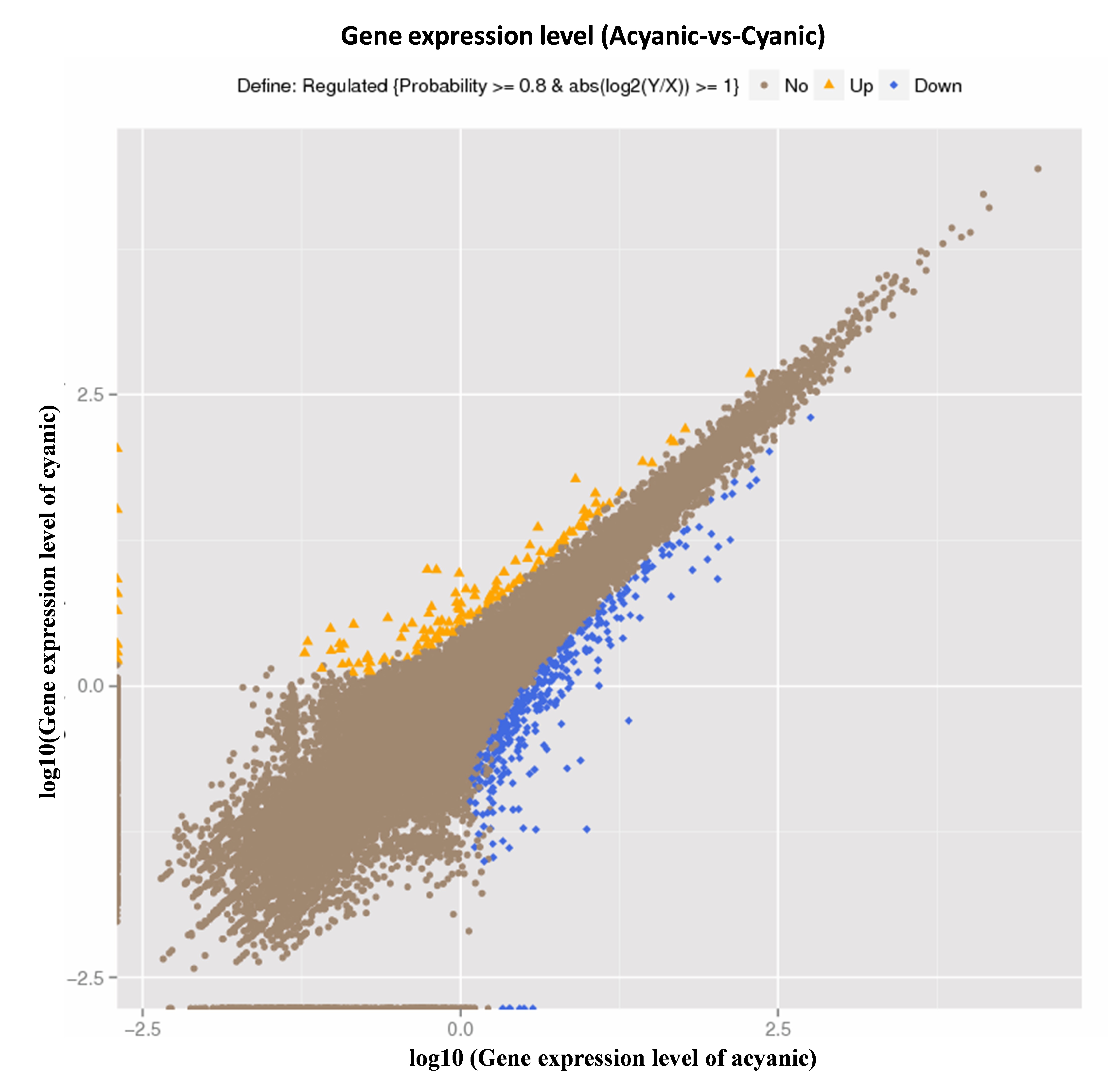

Supplement: Supplementary file 1 — Additional file 1: Figure S1. DEGs between the cyanic and acyanic petals. Transcriptome sequencing was performed with three biological replicates. The parameters “probability ≥0.8” and “log2 ratio ≥1” were used as the thresholds to determine the DEGs. An orange triangle represents the upregulated genes, a green square indicates the downregulated genes, and gray dots indicate the genes that did not change significantly between the two transcriptomes. [file 12870_2020_2428_MOESM1_ESM.jpg]

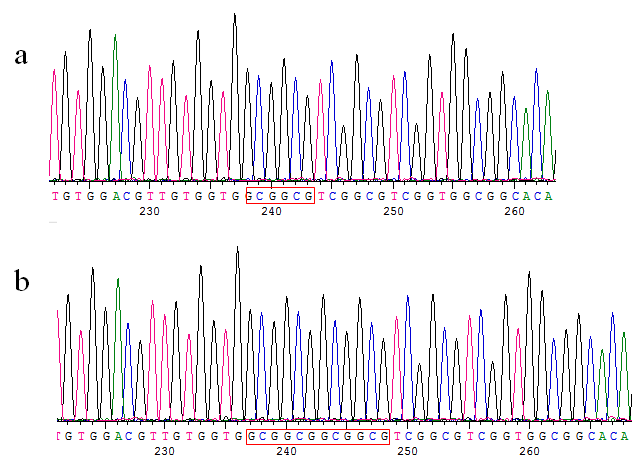

Supplement: Supplementary file 2 — Additional file 2: Figure S2. Validation of the small insertion in the F3’H cDNAs. a. Fragment sequence of the F3’H cDNA in cyanic flowers; b. fragment sequence of the F3’H cDNA in acyanic flowers. [file 12870_2020_2428_MOESM2_ESM.png]

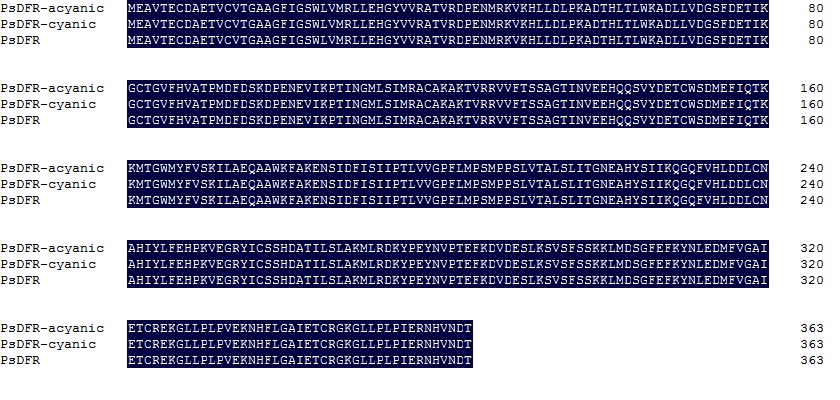

Supplement: Supplementary file 3 — Additional file 3: Figure S3. The alignment of DFR amino acids from cyanic and acyanic branches of ‘Er Qiao.’ PsDFR (GenBank accession number KJ466968) was obtained from Paeonia suffruticosa cultivar ‘Luoyang Hong.’ [file 12870_2020_2428_MOESM3_ESM.jpg]

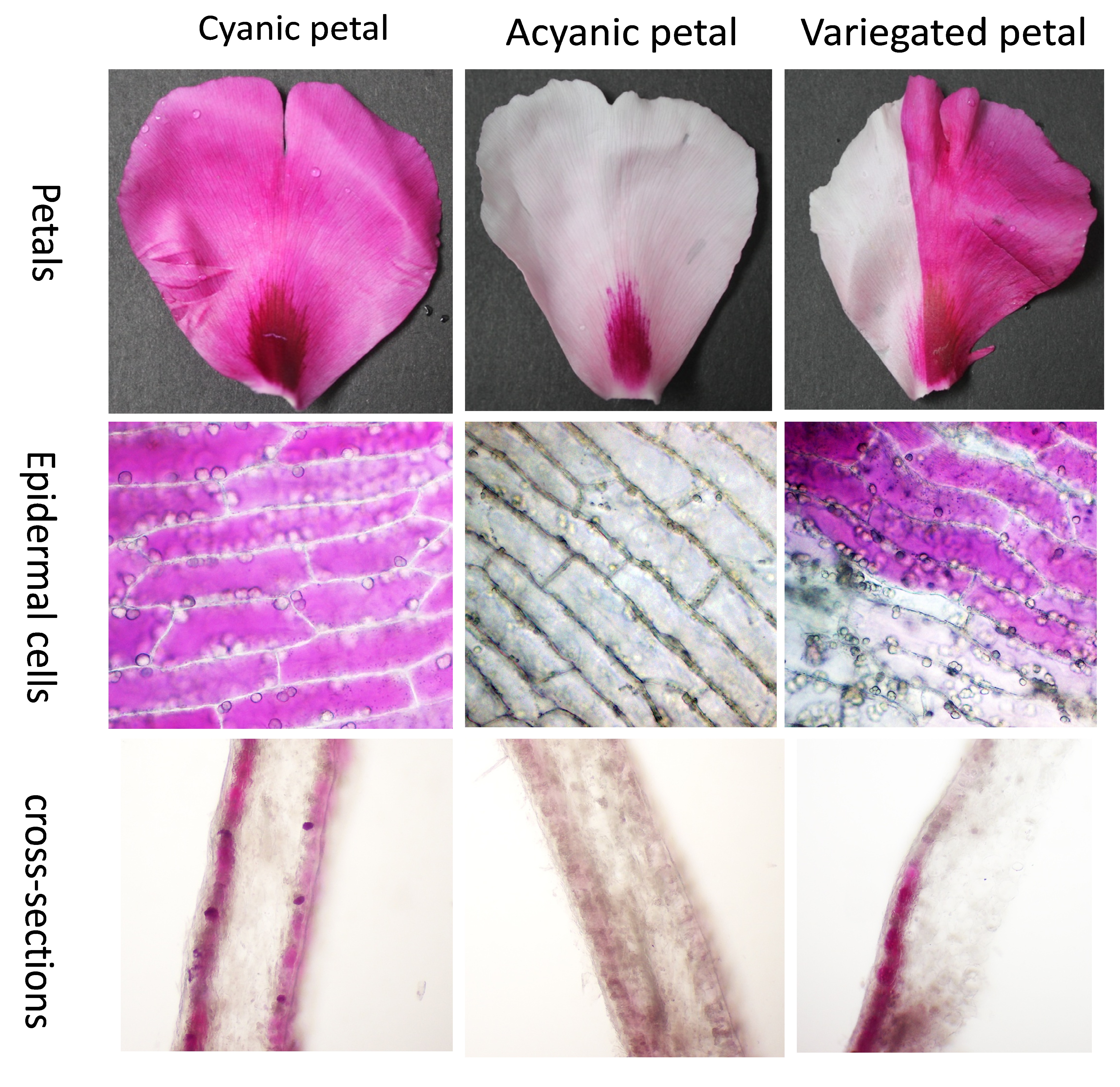

Supplement: Supplementary file 4 — Additional file 4: Figure S4. Examination of anthocyanin accumulation in the petal cell layers. Photos of epidermal cells (original magnification, 400×) and cross-sections (original magnification, 100×) were taken under a microscope. [file 12870_2020_2428_MOESM4_ESM.jpg]
