# Supplementary material for: Tree peony variegated flowers show a small insertion in the F3’H gene of the acyanic flower parts
Source: BMC Plant Biol. 2020 May 12;20:211. doi: 10.1186/s12870-020-02428-x (PMC7216414; doi:10.1186/s12870-020-02428-x)
Supplement: Supplementary file 5 — Additional file 5: Table S1. Statistics of assembly quality. Table S2. KEGG enrichment of the DEGs between cyanic and acyanic flowers. Table S3. Primers used for qRT-PCR. [file 12870_2020_2428_MOESM5_ESM.docx]

**Table S1. Statistics of assembly quality**

|  | Sample | Total Number | Total Length (nt) | Mean length (nt) | N50 |
| --- | --- | --- | --- | --- | --- |
| Transcript | Acyanic_01 | 116,702 | 44,606,550 | 382 | 915 |
|  | Acyanic_02 | 112,988 | 43,630,494 | 386 | 904 |
|  | Acyanic_03 | 108,878 | 42,606,420 | 391 | 929 |
|  | Cyanic_01 | 113,923 | 43,295,370 | 380 | 910 |
|  | Cyanic_02 | 116,011 | 43,952,000 | 379 | 906 |
|  | Cyanic_03 | 114,844 | 43,664,664 | 380 | 909 |
| Unigene | Acyanic_01 | 53,376 | 50,012,503 | 937 | 1513 |
|  | Acyanic_02 | 52,677 | 48,813,248 | 927 | 1481 |
|  | Acyanic_03 | 50,780 | 47,166,575 | 929 | 1482 |
|  | Cyanic_01 | 51,337 | 47,217,430 | 920 | 1477 |
|  | Cyanic_02 | 52,001 | 48,234,456 | 928 | 1489 |
|  | Cyanic_03 | 51,610 | 48,042,272 | 931 | 1507 |
| All-Unigene |  | 75,669 | 80,664,445 | 1066 | 1679 |

**Table S2. KEGG enrichment of the DEGs between cyanic and acyanic flowers**

| Pathway | DEGs with pathway annotation | All genes with pathway annotation | Qvalue | Pathway ID |
| --- | --- | --- | --- | --- |
| Phenylpropanoid biosynthesis | 15 (7.81%) | 319 (1.29%) | 2.40e-06 | ko00940 |
| Ascorbate and aldarate metabolism | 10 (5.21%) | 148 (0.6%) | 9.17e-06 | ko00053 |
| Stilbenoid, diarylheptanoid and gingerol biosynthesis | 9 (4.69%) | 122 (0.5%) | 1.17e-05 | ko00945 |
| Cutin, suberine and wax biosynthesis | 8 (4.17%) | 96 (0.39%) | 1.52e-05 | ko00073 |
| Biosynthesis of secondary metabolites | 44 (22.92%) | 2729 (11.08%) | 2.81e-05 | ko01110 |
| alpha-Linolenic acid metabolism | 8 (4.17%) | 147 (0.6%) | 2.42e-04 | ko00592 |
| Flavonoid biosynthesis | 9 (4.69%) | 196 (0.8%) | 2.45e-04 | ko00941 |
| Fatty acid elongation | 5 (2.6%) | 47 (0.19%) | 2.81e-04 | ko00062 |
| Diterpenoid biosynthesis | 6 (3.13%) | 85 (0.34%) | 4.22e-04 | ko00904 |
| Limonene and pinene degradation | 6 (3.13%) | 97 (0.39%) | 7.95e-04 | ko00903 |
| Cyanoamino acid metabolism | 6 (3.13%) | 125 (0.51%) | 2.87e-03 | ko00460 |
| Isoquinoline alkaloid biosynthesis | 4 (2.08%) | 49 (0.2%) | 3.36e-03 | ko00950 |
| Flavone and flavonol biosynthesis | 5 (2.6%) | 102 (0.41%) | 6.64e-03 | ko00944 |
| Metabolic pathways | 58 (30.21%) | 5280 (21.43%) | 1.33e-02 | ko01100 |
| Isoflavonoid biosynthesis | 3 (1.56%) | 40 (0.16%) | 1.74e-02 | ko00943 |
| Inositol phosphate metabolism | 5 (2.6%) | 160 (0.65%) | 3.71e-02 | ko00562 |
| Tyrosine metabolism | 5 (2.6%) | 167 (0.68%) | 4.15e-02 | ko00350 |
| Starch and sucrose metabolism | 10 (5.21%) | 546 (2.22%) | 4.21e-02 | ko00500 |

**Table S3. Primers used for qRT-PCR**

| Gene | Forward Primer（5‘-3’） | Reversed Primer（5‘-3’） |
| --- | --- | --- |
| PsCHS1 | AGCAGAGAACAACAAAGGGTCACG | TCAGCACCGACAATAACCGCAG |
| PsCHI1 | AAATTCCCACCTGGTTCTTCTATTC | CTCCTTTGACCTTATCCATCCTTCA |
| PsF3H1 | CCCAAGGTAGCCTACAACCAA | GAAAATCCCCCAGTCTTCACA |
| PsF3’H | AACTTGTTCACGGCAGGGAC | GAAGGGGAGATTTGGGAGGT |
| PsDFR | AGCCAACAATAAATGGAATG | GGATCTTGGACACGAAATAC |
| PsANS | TAGAACAAGAAGTCGGTGGC | GTGGAGGATGAAAGTGAGGG |
| Ps3GT | ACTGATTTCATCCGAGACAAGG | TGGAGTTCAAGTTTCCGACG |
| PsGST | CAAAGGTTGTCAAAGAGCAGC | TTCACATTCTTCCTCACGGTC |
| PsMATE | ATGGACAGGAATGATTGGAGG | TTGGTTCAATGGGTCGTGTT |
| PsMRP | TCAGTTTGAATGTTTTGAGGAGG | AGAAGAGCGTAGACCAGATTGTAGT |
| PsFLS1 | AGTGCAAGCAAAGAATGGGG | TCTCTTCCAATGGCAGCTCA |
| PsFLS2 | TATTACCCACCTTGCCCTCG | TGAAGCCCTTGGACCTCATT |
| Ubiquitin | GACCTATACCAAGCCGAAG | CGTTCCAGCACCACAATC |
| GADPH | ACGTTGTTGAGTCCACTGGA | ACGACAAACATGGGTGCATT |
|  |  |  |
